# Effect of long-term paired associative stimulation on the modulation of cortical sensorimotor oscillations after spinal cord injury

**DOI:** 10.1038/s41394-022-00506-w

**Published:** 2022-04-05

**Authors:** Jukka Vanhanen, Lauri Parkkonen, Jyrki P. Mäkelä, Alexandra Tolmacheva, Anastasia Shulga, Andrey Rodionov, Erika Kirveskari

**Affiliations:** 1 BioMag Laboratory, HUS Diagnostic Center, Helsinki University Hospital and University of Helsinki, Helsinki, Finland; 2 HUS Diagnostic Center, Clinical Neurophysiology, Clinical Neurosciences, Helsinki University Hospital and University of Helsinki, Helsinki, Finland; 3 Department of Neuroscience and Biomedical Engineering, Aalto University, Espoo, Finland; 4 Clinical Neurosciences, Neurology, Helsinki University Hospital and University of Helsinki, Helsinki, Finland

**Keywords:** Trauma, Cortex, Spinal cord diseases

## Abstract

**Study design:**

A prospective interventional case series.

**Objectives:**

To explore changes in the modulation of cortical sensorimotor oscillations after long-term paired associative stimulation (PAS) in participants with spinal cord injury (SCI).

**Setting:**

BioMag Laboratory, HUS Diagnostic Center, University of Helsinki and Helsinki University Hospital, Helsinki, Finland.

**Methods:**

Five patients with chronic incomplete SCI received unilateral spinal PAS to upper limb for 16–22 days. Changes in the modulation of sensorimotor oscillations in response to tactile stimulus and active and imaginary hand movements were assessed with magnetoencephalography recorded before and after the intervention.

**Results:**

PAS restored the modulation of sensorimotor oscillations in response to active hand movement in four patients, whereas the modulation following tactile stimulation remained unaltered. The observed change was larger in the hemisphere that received PAS and preceded the clinical effect of the intervention.

**Conclusions:**

Long-term spinal PAS treatment, which enhances the motor functions of SCI patients, also restores the modulation of cortical sensorimotor oscillations.

## Introduction

Spinal cord injury (SCI) has a devastating and long-lasting, even permanent effect on an individual, warranting extensive rehabilitation. Neuromodulation is emerging as a promising intervention for SCI patients. Epidural electrical spinal cord stimulation (EES) has proven beneficial in several trials [1–3]. Our group has recently reported motor improvement of patients with chronic incomplete SCI treated with long-term paired associative stimulation (PAS) [4–8]—the combination of transcranial magnetic stimulation (TMS) with simultaneous peripheral nerve stimulation (PNS) [9, 10]. The physiological mechanisms of these interventions on SCI, however, remain incompletely understood.

Besides the physical disruption of the corticospinal axons and spinal neurons in SCI, the ensuing neurological sequelae are also due to pathophysiological changes in the spinal cord circuits following the injury [11, 12]. Such disturbances progress in the central nervous system beyond the initial SCI, resulting in changes even at the cortical level. For example, SCI alters the dynamics of the sensorimotor oscillatory activity in the brain [13, 14]. The level of this activity normally diminishes in response to motor activation or somatosensory stimulation, leading to suppression (or event-related desynchronization) of the oscillatory activity, and transiently increases after the cessation of the movement or somatosensory stimulation, known as a rebound (or event-related synchronization) [15, 16]. In SCI patients, the rebound amplitude after an attempted movement is decreased, and the magnitude of this change correlates with the severity of SCI [14]. A recent study on SCI patients reported that EES during an attempted movement led to an increased rebound of the sensorimotor oscillations [1], but studies examining these oscillations in long-term PAS interventions are lacking.

This prospective case series explored the dynamics of sensorimotor oscillations in patients with chronic incomplete SCI receiving PAS. We recorded magnetoencephalography (MEG) to study the cortical dynamics before and after the PAS intervention. Our aim was to find out how PAS affects the modulation of cortical sensorimotor oscillations, and whether it correlates with the clinical outcome of long-term PAS.

## Methods

### Patients

This case series comprised five SCI patients treated with long-term PAS: four patients after traumatic tetraplegia (Trial 1) [5] and one patient after nontraumatic tetraplegia (Trial 2) [6]. In Trial 1, patients underwent 16 PAS sessions in 4 weeks (five sessions a week for 2 weeks and three sessions a week for 2 weeks). The fifth patient, from Trial 2, received 22 PAS sessions (five sessions a week for 2 weeks and three sessions a week for 4 weeks).

### Paired associative stimulation (PAS)

The patients from Trial 1 received PAS (a combination of PNS to the upper limb and TMS to the contralateral hemisphere) to a randomly chosen side, and, as a control, PNS combined with sham TMS to the other side. A plastic block inserted between the TMS coil and scalp enabled the delivery of sham stimulation with the patient blinded to the type of stimulation. The researcher administering TMS was aware of the stimulation type. Patient 5 from Trial 2 received PAS to the weaker side, with no stimulation to the contralateral side. During the stimulations, the patients were instructed to concomitantly imagine the stimulus-induced movements. For all patients, the intervention was administered to three targets: median nerve and the corresponding contralateral cortical motor hotspot of abductor pollicis brevis muscle, ulnar nerve, and the hotspot of abductor digiti minimi, and radial nerve and the hotspot of brachioradialis muscle.

TMS and PNS were timed to produce coinciding neuronal impulse volleys at the corticomotoneuronal synapses in the spinal cord [17, 18]. A biphasic figure-of-eight coil with magnetic resonance imaging–guided navigation (eXimia magnetic stimulator, Nexstim Ltd., Helsinki, Finland) was used for TMS, and a Dantec Keypoint device (Natus Medical Inc., Pleasanton, CA, USA) for PNS. Both trials shared the same TMS parameters. The PNS frequency was 50 Hz in Trial 1 and 100 Hz in Trial 2 (see Table 1 for additional details).

**Table 1:** Details of Trial 1 and Trial 2.

	Trial 1 [5]	Trial 2 [6]
Number of patients for this study	4	1
PAS sessions	5 + 5 + 3 + 3 = 16	5 + 5 + 3 + 3 + 3 + 3 = 22
PAS hand selection	At random	Weaker hand
Contralateral hand	PNS combined with sham TMS	No stimulation
PNS trains	6 pulses at 50 Hz	6 pulses at 100 Hz
PAS targets	Median nerve—APB hotspot, Ulnar nerve—ADM hotspot, Radial nerve—Brachioradialis hotspot	
TMS parameters	20 min at 0.2 Hz, 100% SO (a total of 240 pulses)	
TMS-PNS interval	Minimum F latency minus MEP latency [18]	

*APB* abductor pollicis brevis muscle, *ADM* abductor digiti minimi muscle, *SO* stimulator output, *MEP* motor evoked potential.

### Clinical evaluation

A physiotherapist, blinded to the type of the treatment, evaluated the motor function of each patient using the Daniels and Worthingham’s muscle test on a 0–5 scale [19] before, immediately after, and one month after the PAS intervention (see Table S1 for detailed results of the muscle tests). The mean difference of motor scores between the first and second evaluation of each tested muscle was taken to represent the immediate effect of PAS, and the mean difference between the first and third evaluation the long-term effect of PAS.

### Magnetoencephalography (MEG)

MEG (306-channel TRIUX^TM^ system, MEGIN Oy, Helsinki, Finland) was recorded 5–25 days before the PAS intervention and 0–6 days after the last stimulation. Electro-oculogram (EOG) and electromyogram (EMG) of wrist flexors in both forearms were recorded as well. Head position in the MEG helmet was tracked continuously during the measurement by five indicator coils attached to the head and digitized prior to the measurement with anatomical landmarks. The MEG sampling rate was 1000 Hz.

We used three different conditions to assess the sensorimotor system: active movement paradigm, imaginary movement paradigm and tactile stimulation.

In the active movement paradigm, patients held a plastic bottle in their hand and, in response to auditory cues, grasped it repeatedly for 2 s with a 4-s resting interval. We monitored the EMG signal to assure that the patient performed the grasp correctly and recorded ~100 cycles of contraction and relaxation for both hands.

The imaginary movement paradigm was similar to the active paradigm, except that instead of real grasping, the patient imagined grasping with the tested hand.

Tactile stimuli were generated by plastic diaphragms inflated by compressed air (Somatosensory Stimulus Generator, 4-D NeuroImaging Inc., San Diego, CA, USA). They were delivered to the left and right index fingers alternatingly at 4-s intervals. About 50 responses were recorded for each side.

The researcher analyzing the MEG recordings was unaware of the side of the PAS-treated hand in each patient. The MEG data were inspected visually and channels with excessive noise or artifacts were omitted. Thereafter, the raw data were processed with the spatiotemporal signal space separation method [20] using a correlation window of 16 s implemented in the MaxFilter® software version 2.2 (MEGIN Oy, Helsinki, Finland) to reduce the effect of external artifacts. In addition, to enable sensor-level comparison between the longitudinal measurements, the post-intervention measurement was transformed to the same head position as the pre-intervention measurement in each patient. Finally, the data were low-pass-filtered to 111 Hz and downsampled to 333 Hz.

MEG data were analyzed further using custom scripting [21] of the MNE-Python software package [22, 23]. First, the MEG signals were high-pass filtered at 1 Hz and independent component analysis was used to suppress ocular artefacts; the independent components correlating most with the EOG signal were removed. The data were split into epochs around each stimulus onset and epochs with prominent artifacts were manually excluded.

To assess the temporal dynamics of oscillatory activity, a time–frequency representation (TFR) was computed for each epoch in the frequency range 5–30 Hz (Morlet wavelets with length adjusted for each frequency band). A baseline correction was applied by subtracting the power of each frequency during the baseline period (800–200 ms before the stimulus onset) from the epoch. The magnitudes of the TFR epochs were then averaged. The gradiometer channel around the rolandic area with the most prominent modulation was selected for further analysis from both hemispheres.

Subtraction of the pre-intervention TFR from the post-intervention TFR enabled visualization of the changes in oscillatory modulation. The statistical significance of these changes was determined with a cluster permutation test [24] using an alpha-level of 0.05.

For quantification of the changes in oscillatory power, the dynamics of the frequency with most prominent change after the intervention were plotted, and the relative rebound strength was calculated by dividing the maximum rebound power with the baseline oscillatory power.

Cortical sources of the somatosensory evoked fields (SEF) elicited by tactile stimuli were estimated by fitting current dipoles to the peaks of the responses (“xfit” software; MEGIN Oy, Helsinki, Finland). Separate equivalent current dipoles (ECD) were estimated for the activation of primary somatosensory cortex (S1) and secondary somatosensory cortices (S2). We hypothesized that the location of the ECD should not change, and that PAS would primarily affect the dipole strength (Q).

To meaningfully compare pre- and post-intervention dipole strengths, we sought for a fixed dipole position that explained the responses of both measurements. First, the epochs of both measurements were combined and averaged in MNE-Python. Then, an ECD was fitted to this averaged data: for the S1, the maximum activation was sought from the channels around rolandic area contralateral to the stimulated hand. For the S2 activation, the ECD was sought from the opercular region contralateral (S2c) and ipsilateral (S2i) to the stimulated hand.

The ECD strengths were then estimated separately for the pre- and post-intervention measurements; the ECD strength was fitted to these data with the fixed dipole location and orientation parameters obtained previously. The goodness-of-fit value was scrutinized to ensure that the ECD was an appropriate model for both measurements.

## Results

All patients underwent the intervention as intended and, as previously reported, improved on their motor function (Tables 2 and S1) [5, 6].

**Table 2:** Patient characteristics and clinical results of the PAS intervention.

Patient	Age	Sex	Etiology	Time since events	Neurological level	AIS	Change of motor scores			
							Immediately after PAS		1 month after PAS	
							PAS side	Contralateral side	PAS side	Contralateral side
1	38	Male	Traumatic	4 years 3 months	C7	B	1.1	0.7	1.3	0.8
2	38	Male	Traumatic	5 years 10 months	C7	D	1.2	0.4	1.3	0.5
3	42	Male	Traumatic	6 years 7 months	C4	C	0.6	0.8	1.2	1.0
4	53	Male	Traumatic	3 years 11 months	C3	C	0.2	0.4	0.4	0.2
5	49	Male	Spondylodiscitis, epidural abscess C2-T2	1 year 3 months	C3	D	2.1	1.3	2.4	1.7

Change of motor scores represents the mean difference of motor scores of all tested muscles between the pre-intervention and immediate post-intervention assessments, and between the pre-intervention and 1-month follow-up assessments. The clinical results have been previously published [5, 6].

*SCI* spinal cord injury, *AIS* American Spinal Injury Association Impairment Scale, *PAS* paired associative stimulation.

### Active movement

**Fig. 1:**
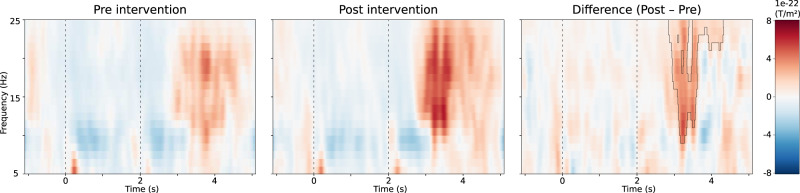
Time–frequency representation of the movement-induced oscillatory modulation in the hemisphere contralateral to the hand movement. The muscle contraction starts at 0 s and ends at 2 s. Example of Patient 5, before and after the intervention. Illustration on the right represents the difference (post- minus pre-intervention), and the black contour outlines the statistically significant cluster of difference (*p* < 0.05).

**Fig. 2:**
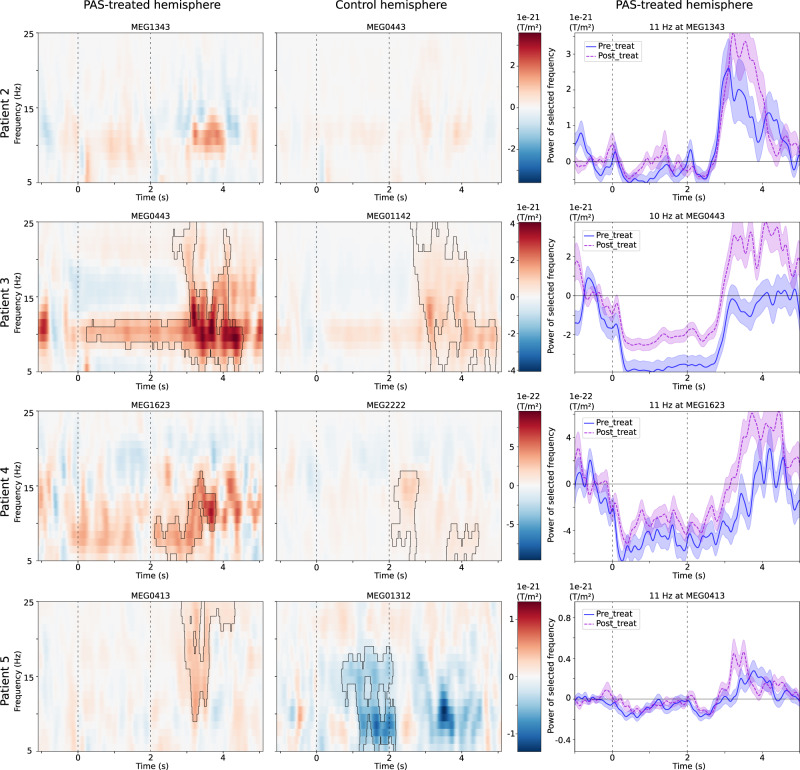
Modulation of sensorimotor oscillations in response to contralateral hand muscle contraction, beginning at 0 s and ending at 2 s (dashed vertical lines). Left and middle columns: time–frequency representations of the difference between the pre- and post-intervention: positive values (in red color) indicate stronger oscillatory power in the post-PAS measurement. Significant clusters of difference (*p* < 0.05) are outlined. Right column: modulation of the frequency with the largest change between the two measurements (lines represent the average power of epochs, and shaded areas the standard error of mean).

In the active movement paradigm, the most prominent modulation of the sensorimotor oscillations appeared in the hemisphere contralateral to the hand engaged in the task and was located around the rolandic area. In the ipsilateral hemisphere, a similar but weaker modulation was detected. The active movement paradigm measurement after the PAS intervention failed in Patient 1 due to technical reasons. In Patients 2–5, the rebound of the sensorimotor oscillations following contralateral hand movement was stronger after the intervention than before it in the PAS-treated hemisphere, and this difference was statistically significant in 3 out of the 4 patients (Table 3a and Figs. 1, 2). In three patients, the rebound increased in the control hemisphere as well, but always less than in the PAS-treated hemisphere.

**Table 3:** The relative strength of sensorimotor oscillatory rebound (maximum rebound power divided by baseline oscillatory power).

Patient	Treatment (Hemisphere)	Rebound strength (%)		Change in rebound strength (%)	Significant cluster (*p* value)
		Pre-intervention	Post-intervention		
a. After contralateral hand movement					
1	PAS (Right)	106	N/A	N/A	N/A
	Sham (Left)	66			
2	PAS (Right)	370	503	36	No
	Sham (Left)	238	287	21	No
3	PAS (Left)	5	112	2140	Yes (0.003)
	Sham (Right)	79	229	190	Yes (0.001)
4	PAS (Left)	29	73	152	Yes (0.005)
	Sham (Right)	31	65	110	Yes (0.032)
5	PAS (Left)	83	153	84	Yes (0.002)
	No treatment (Right)	107	31	−71	No
b. After imaginary movement of the contralateral hand					
1	PAS (Right)	73	N/A	N/A	N/A
	Sham (Left)	73			
2	PAS (Right)	297	124	−58	Yes (0.003)
	Sham (Left)	224	82	−63	No
3	PAS (Left)	51	110	116	No
	Sham (Right)	52	161	210	No
4	PAS (Left)	26	67	158	Yes (0.002 and 0.008)
	Sham (Right)	32	31	−3	No
5	PAS (Left)	45	57	27	No
	No treatment (Right)	3	29	867	No
c. After tactile stimulus of the contralateral hand					
1	PAS (Right)	27	46	70	No
	Sham (Left)	75	70	−6	No
2	PAS (Right)	80	88	1	No
	Sham (Left)	117	149	27	No
3	PAS (Left)	70	72	3	No
	Sham (Right)	63	94	49	No
4	PAS (Left)	No modulation	No modulation	N/A	N/A
	Sham (Right)	No modulation	No modulation		
5	PAS (Left)	N/A	167	N/A	N/A
	No treatment (Right)		83

The clinical assessment performed immediately after the intervention did not reflect the enhanced rebound on PAS-treated hemisphere: motor scores of two patients improved more on the control than PAS-treated side (Table 2). At the 1-month evaluation, however, the clinical assessment paralleled the changes seen in oscillatory modulation after the intervention: motor scores of each patient improved more on the PAS-treated than control side (median motor-score change +1.3 vs. +0.8, respectively). The median increase of the rebound was +118% in the PAS-treated hemisphere and +66% in the control hemisphere. The EMG signal revealed that Patient 2 had difficulties in maintaining consistent timing of the movement in the pre-PAS measurement, resulting in increased variance of the responses.

### Imaginary movement

Imaginary movement produced a robust suppression and rebound of the sensorimotor oscillations, but visual analysis revealed that in comparison with the active-movement paradigm the modulation was more bilateral.

The rebound was significantly stronger in the PAS-treated hemisphere in Patient 4 after the intervention. In Patient 2, the rebound was significantly weaker in the PAS-treated hemisphere after the intervention. No significant changes occurred in Patients 3 and 5 (Table 3b). The post-intervention imaginary paradigm measurement failed in Patient 1.

### Tactile stimulus-induced oscillatory modulation

Tactile stimuli induced a detectable modulation of sensorimotor oscillations in three patients (Patients 1–3). The rebound did not change significantly in any of the patients (Table 3c). The suppression following tactile stimulus was significantly stronger in the PAS-treated hemisphere after the intervention in two patients (Patient 1 and 3). The pre-intervention measurement with tactile stimuli failed in Patient 5.

### Somatosensory evoked fields (SEFs)

S1 and S2 responses were identified consistently in Patients 1–3. The ipsilateral S2 response to the left-hand stimulation was not detected in Patient 3. No SEFs were detected in Patient 4. No significant changes were seen in the latencies or dipole moments of the responses between pre- and post-intervention measurements (Table 4).

**Table 4:** Peak latencies and dipole moments (Q) of the sources of somatosensory evoked fields (SEFs).

Patient	Stimulated hand	S1			S2c			S2i		
		Peak latency [ms]	Pre: Q [nAm] (GOF [%])	Post: Q [nAm] (GOF [%])	Peak latency [ms]	Pre: Q [nAm] (GOF [%])	Post: Q [nAm] (GOF [%])	Peak latency [ms]	Pre: Q [nAm] (GOF [%])	Post: Q [nAm] (GOF [%])
1	Left*	58	26 (96)	24 (97)	103	68 (93)	65 (89)	131	19 (93)	26 (91)
	Right	52	28 (93)	26 (92)	94	53 (96)	62 (97)	117	84 (95)	84 (96)
2	Left*	64	33 (95)	43 (91)	122	99 (95)	71 (83)	148	88 (94)	98 (83)
	Right	64	70 (91)	68 (91)	129	89 (92)	83 (92)	135	54 (93)	46 (67)
3	Left	55	34 (94)	38 (95)	88	61 (74)	54 (81)	102	49 (93)	34 (79)
	Right*	54	28 (92)	28 (88)	80	108 (88)	129 (82)	120	Absent	Absent
4	Left	Absent			Absent			Absent		
	Right*	Absent			Absent			Absent		
5	Left	N/A			N/A			N/A		
	Right*									

S1 refers to responses from primary somatosensory cortex, S2c and S2i to responses from secondary somatosensory cortices contralateral and ipsilateral to the stimulus, respectively. Peak latency is from the pre-intervention MEG. *PAS-treated hand.

*GOF* goodness-of-fit value, *N/A* not applicable.

## Discussion

This case series explored the effect of long-term PAS on cortical sensorimotor oscillations in SCI patients. The main finding was that the rebound amplitude of sensorimotor oscillations following active movement increased after the intervention, and this increase was more prominent in the PAS-treated hemisphere. This adds to the evidence that PNS alone does not produce as good motor improvement as PAS [4, 5]. Our finding also suggests that assessing the modulation of sensorimotor oscillations after PAS intervention could predict the ultimate clinical PAS effect. In addition, this study clarifies some immediate neurophysiological effects of a long-term neuromodulation in patients with SCI.

As previously reported, our long-term PAS intervention was beneficial, and motor scores of all patients improved during the 1-month follow-up, more so on the PAS-treated side [5, 6]. The changes observed in movement-induced oscillatory rebound appeared to correlate with the clinical effect of the PAS, as the rebound increased more in the PAS-treated hemisphere. This enhancement of rebound is in line with a previous study where an EES intervention resulted in a similar effect [1]. As SCI results in a weakening of the oscillatory rebound [14], the restoration of this cortical activity presumably represents a sign of recovery. This notion is further supported by our finding that clinical improvement paralleled the enhanced rebound. Considering the association of enhanced rebound and motor improvement, a general predictive model between these factors would be intriguing, but due to the small sample size, we could not establish such a model.

The regained rebound after PAS could merely be a secondary reflection of the improved motor function. Immediately after the PAS intervention, however, the motor scores of two patients improved more on the control side receiving PNS only. Contrary to this early clinical presentation, the increase in oscillatory rebound was evident in all patients in the PAS-treated hemisphere, and was associated with the impending motor improvement, observed at the 1-month evaluation.

López-Larraz et al. reported that in subacute SCI, the clinical improvement was linked with preservation of oscillatory suppression, which could be a marker of the clinical outcome [13]. The longitudinal evolution of oscillatory rebound has not been reported in SCI patients. In patients with stroke, the rebound magnitude correlated positively with clinical outcome [25]. The oscillatory rebound has been suggested to reflect the excitability or active inhibition of the sensorimotor cortex and it could be involved in the control of voluntary movements [15, 26, 27]. We speculate that the increased rebound observed after the PAS intervention reflects restoration of excitation–inhibition balance in the sensorimotor cortex. This could, in turn, promote the impending improvement of motor control. Thus, the restoration of rebound could be considered as an early marker of the motor recovery process.

In most patients, the oscillatory modulation was increased after the therapy in the sham-treated hemisphere as well. Studies with therapeutic TMS interventions often report notable placebo effects [28]. Due to our study setup, we could not differentiate whether this rebound increase was due to a placebo effect of the sham intervention, or resulted from the activation of the strong interhemispheric connections between the sensorimotor cortices.

The observed oscillatory changes in this study focused on the α-frequency band, and in three patients, the changes extended to the β-band as well. We chose not to confine our analysis to predefined α (8–13 Hz) or β (14–30 Hz) frequency bands, as the frequencies of sensorimotor rhythms can change over time in SCI patients [13]. Furthermore, no prior data existed to support the notion that spinal PAS would selectively affect oscillations of a specified frequency band. A subgroup analysis regarding different frequency bands and treatment outcome was unreasonable because of the small sample size.

Considering that the patients engaged in motor imagery during the PAS delivery, we hypothesized that the imaginary paradigm in MEG would accurately reveal the possible changes in oscillatory modulations. Although the oscillatory rebounds were stronger after the intervention in 3 out of 4 patients, this change was significant in only one patient. This probably reflects the high variability in the execution of the imagery task. Even in healthy subjects the performance of movement imagery is variable [29], and any disturbance in the sensorimotor system is likely to further increase this variability.

Although the cortical primary sensory and motor areas are highly interconnected and the oscillatory modulations following sensory and motor stimuli are usually related, these two systems behaved differently after the intervention. The enhancement of rebound detected in the motor task was lacking in the tactile condition. In line with this, SEFs remained unaltered. Accordingly, PAS did not improve somatosensation in our patients [5], and other studies targeting specifically motor pathways by paired stimulation of M1 and lower motor neurons have reported similar results [4, 7, 8, 30]. Of note, in Patient 4, the oscillatory modulation following a tactile stimulus as well as SEFs were completely absent, in accordance with a clinical status of no somatic sensation of the index fingers; a motor attempt, however, did induce a noticeable modulation of the sensorimotor oscillations.

The coincidence of the peripheral stimulation and TMS pulse can have various consequences depending on the stimulus properties, especially the timing [10]. In our protocol, the stimulus-induced volleys that coincided at the spinal level likely induce LTP-like plasticity in the spinal motor synapses [18, 31]. From this perspective, the lack of change in the oscillatory modulation following sensory stimulation is unsurprising. Analogously, the restoration of movement-induced cortical modulation probably reflects a secondary effect following plastic changes in the spinal cord. The observation that this cortical effect was detectable earlier than the favorable clinical outcome in some patients, however, raises the possibility that cortical plasticity is also involved in the fundamental mechanisms of long-term spinal PAS. Cortical involvement could also explain why both upper extremities tend to improve shortly after one-sided PAS intervention. Moreover, even a direct cortical effect is possible in our spinal PAS protocol [17], but the design of this study prevented differentiating the possible spinal and cortical effects.

A recent study demonstrated that voluntary muscle activation combined with PAS prolongs the PAS-induced motor effect [32]. We aimed to strengthen the PAS effect with concomitant motor imagery, known to activate neural circuits involved in actual motor activation [33, 34]. In addition, such motor imagery might result in a more efficient cortical activation by lowering the motor threshold. The performance of motor imagery is, however, difficult to monitor objectively, and creating a feedback mechanism for this performance is challenging. The high variability seen in the results of the imaginary paradigm suggests that the design of this paradigm needs improvement, and probably the instructions for motor imagery during the PAS require further optimization as well.

The small number of patients in our study is an obvious limitation, and caution is necessary when generalizing the results. One patient also had a slightly different intervention than the others, which increases the heterogeneity of our patient group. On the other hand, similar results in traumatic patients and in the nontraumatic patient suggest that the type of the spinal injury is not decisive for the effect of PAS on sensorimotor oscillations. We did not have a placebo group, although 4/5 patients received a sham treatment to the contralateral side, which served as a within-subject control for the analysis. In the active movement paradigm, the 4-s period of relaxation between consecutive hand movements turned out to be somewhat short in two patients, in whom the oscillatory rebound extended to the baseline period of the following epoch. This did not, however, impinge the comparisons between the pre- and post-intervention measurements as the rebound was similarly long-lasting in both. Finally, some of the MEG measurements failed due to technical reasons.

Albeit small, this is a unique patient series, exploring the mechanisms underlying a state-of-the-art neuromodulation on SCI patients. All our patients underwent a careful examination and follow-up during the study until up to 1 month after the intervention, and all patients adhered to the protocol meticulously. Despite their impaired motor function, all patients were able to perform the instructed tasks during the MEG recordings.

PAS intervention restored the modulation of sensorimotor oscillations, and the enhancement of rebound substantially preceded the clinical improvement. This could provide a means to predict the clinical effect of long-term PAS earlier than achievable with clinical tests and could prove useful in fine-tuning the PAS protocol for individual patients.

## Supplementary information

Supplementary Table 1

## Data Availability

Ethical restrictions imposed by the hospital’s research ethics committee prevent the authors from making brain imaging data publicly available without restrictions, as these data cannot be fully anonymized. However, the relevant summary tables of the data are available from the authors upon reasonable request and with permission of the hospital’s research ethics committee, for researchers aiming to reproduce the results. The code for conducting the analysis is available from the authors upon request.
